# Exploration of Sensory Processing Difficulties among Children Attending Primary School in Denmark

**DOI:** 10.1155/2021/8893345

**Published:** 2021-03-24

**Authors:** Ann Natasja Nielsen, Åse Brandt, Karen la Cour

**Affiliations:** ^1^Department of Public Health, University of Southern Denmark, Odense, Denmark; ^2^REHPA, The National Knowledge Center for Rehabilitation and Palliative Care, Department of Clinical Research, Odense University Hospital, University of Southern Denmark, Odense, Denmark

## Abstract

Current research shows that children with sensory processing (SP) difficulties have limited participation and enjoyment in their daily activities at school. The aim of this study was to explore the prevalence of SP difficulties among Danish children and to explore possible associated factors. Since SP difficulties can affect children's prerequisites for participation in school activities and learning possibilities, this study focused on primary school children. *Method*. The study was designed as a cross-sectional survey. The sample consisted of 1723 children age 5 to 11 years, who were attending Danish public school (45.5% girls, 53.2% boys). The parents or caregivers of the child completed a Short Sensory Profile (SSP) questionnaire and a demographic questionnaire. One-way ANOVA was used to examine differences between girls and boys regarding sports, geographic area, and parental level of education. Chi-square analysis was used to explore the relationship between sex and SPP scores in the different behavioral sections. Logistic regression analysis was used to investigate possible associations between SP difficulties and sex and the included demographics. *Results.* A total of 21.3% of the children had SSP scores suggesting SP difficulties. Boys had a higher probability of having SP difficulties than girls (odds ratio (OR) = 1.55, confidence level (Cl): 1.22, 1.97). An association was found between participating in sports outside of school and SP difficulties (OR = 0.55, Cl: 0.47, 0.65 (*p* ≤ 0.001)). Additionally, a slight association between SP difficulties and parental education level (OR = 0.80) was found. No association was found regarding geographic area, i.e., where in Denmark the children attended school (OR = 1.00). *Conclusion.* The study results suggest that approximately 20% of the children in Danish public schools might have SP difficulties and over 20% might be at risk of having SP difficulties. The results suggest that Danish schools should focus on both identifying children with SP difficulties and implement interventions such as sensory integration through occupational therapy to help children with SP difficulties, in order to improve their ability to participate and learn from school activities.

## 1. Introduction

Previous studies have shown that children with sensory processing (SP) difficulties have limited participation (defined as the children's involvement in life situations) in and lower levels of enjoyment in everyday activities at school and at home [1, 2]. SP refers to the ability to regulate and organize responses to sensory information in an adaptive and graded manner [3].

Play and school activities are important for children, and their ability to participate in these activities forms a basis for their development [4]. Children with SP difficulties have difficulties responding appropriately and adapt to different sensory information during activities, hereby adversely affecting their participation and potentially their development [3]. Bart et al. found that children with SP difficulties had significantly higher levels of anxiety and ritual behaviors than other children. Furthermore, Bart et al. suggested that ritual behavior may be a coping mechanism for children with anxiety and SP difficulties [5]. Other studies suggest that there is a relationship between childhood SP difficulties and adult anxiety disorders or obsessive-compulsive symptoms [6, 7], as well as an association between SP difficulties and specific language impairment in children [8, 9]. Moreover, it has been shown that children are prone to experience SP difficulties if they were born preterm [10], have Tourette's syndrome [11], or have asthma [12]. Similarly, children with atopic dermatitis and allergic rhinitis have a higher degree of sensory sensitivity which affects their daily activities and choice of leisure activities [13, 14]. Additionally, children with autism [15–18] and attention deficit hyperactivity disorder [19–21] are more likely to struggle with SP difficulties when compared to children who do not suffer from these disorders.

Studies focusing on children without other related diagnoses are limited. Occupational therapy scholars assume a correlation between physical activities and SP [3] [22], since children's SP abilities are dependent on both their ability to participate in play and physical activity; on the other hand, it is through play and physical activity that children develop SP abilities [3, 22].

Previous studies have indicated that boys and girls experience SP difficulties differently [1, 23]. Engel-Yeger found that girls with SP difficulties, when compared to same-aged girls without SP difficulties, chose to participate in different activities, whereas boys with SP difficulties to a higher degree chose to participate in the same activities as boys without SP difficulties [23]. Román-Oyola and Reynolds examined the prevalence of SP difficulties in preschool children in Puerto Rico focusing on socioeconomics as a possible influencing factor. They found that 20% of the children had SP difficulties but found no significant link between income and SP difficulties [24].

It is estimated that 5-16% of children entering preschool in the United States of America struggle with SP difficulties [25]. The prevalence of SP difficulties among children in Scandinavia is unknown. In Denmark, 80% of children start attending public school when they are between 5 and 6 years of age [26]. If young children at risk of having SP difficulties are identified, these difficulties can potentially be addressed through early interventions. A systematic review of occupational therapy for children with SP difficulties found positive effects on children's SP ability, concentration, social skills, reading skills, participation in active play, and belief in their own ability to meet individual goals [27]. Early intervention is economically sensible and could have a great impact on the future education for the children; therefore, the present study focused on children attending primary school [28].

The aim of the present study was to explore the prevalence of SP difficulties among Danish schoolchildren, since SP difficulties are thought to influence the children's participation in school activities [2]. The study additionally, without considering related diagnoses, is aimed at exploring possible associations between SP difficulties and the following demographic characteristics: sex, participation in sports, geographical area, and parental level of education.

## 2. Methods

The study was designed as a cross-sectional study and used a survey to explore the prevalence of SP difficulties among Danish primary schoolchildren.

For the purpose of this study, Danish school grades have been grouped as follows: 0-3 grade (primary), 4-6 grade (middle), and 7-9 grade (end). The present study focuses on children in the primary group, attending 0-3 grade.

### 2.1. Participants

Parents of children attending public school, thereby, the children's possible diagnoses were not considered. In Denmark, children with major special needs due to diagnoses or birth defects attend special classes and schools and were thus excluded from the present study. Participants were recruited over a period of two months through bulletins on the school's private network for parents and the school. The bulletins were in Danish and invited parents of children in grades 0-3 to participate in the survey. The participants were informed that participation was voluntary and anonymous and could be terminated at any time. As an incentive to participate, parents who chose to participate were invited to enter a lottery with prizes of toy store gift certificates.

### 2.2. Procedures

A total of 94 out of 98 municipalities in Denmark were invited to participate; the remaining four municipalities were excluded because they were too small for data to be properly anonymized. Of the 94 municipalities, 12 agreed to participate in the study. The 12 participating municipalities were thought to be representative of Denmark; due to their geographic diversity and large population, ranging from approximately 12.350 to 350.000 citizens. The municipalities who declined the invitation to participate in the study gave different reasons, the most common being that they were in the process of completing surveys themselves or that they had policies not to let researchers contact parents through the school.

### 2.3. Materials

The survey included the Danish version of the *Short Sensory Profile* (SSP) questionnaire to investigate the children's SP ability. SSP is a screening tool completed by a child's parent(s) or caregiver; they are asked to answer 38 items regarding how often they observe their child demonstrating a certain sensory-related behavior. Each item is scored on a 5-point Likert scale (1 = always, 2 = frequently, 3 = occasionally, 4 = seldom, and 5 = never) [29]. The SSP was created for screening programs and research and is a short version of the 125-item *Sensory Profile* questionnaire. In order to best suggest which sensory systems might be interfering with the child's activity performance [29], the 38 SSP items are grouped into seven different behavioral sections: *tactile sensitivity* (7 items), *taste/smell sensitivity* (4 items), *movement sensitivity* (3 items), *underresponsive/seeks sensations* (7 items), *auditory filtering* (6 items), *low energy/weak* (6 items), and *visual/auditory sensitivity* (5 items). The total SSP scores can range from 38 to 190, whereas a score between 155 and 190 indicates a normal performance (between -1 and 1 standard deviation (SD) from the mean), a score between 142 and 154 (±1 and ±2 SD from the mean) indicates a probable difference in SP performance, and a score of 38-141 (scores at 2 SD or more from the mean) indicates a definitive difference in SP performance, indicating SP difficulties. Reliability testing of the *Sensory Profile* and testing of its internal consistency, calculated with Cronbach's coefficient alpha, resulted in values ranging from .47 to .91 [29]. Content validity of the *Sensory Profile* was established during development, using a literature review and expert review, by eight therapists experienced in applying sensory integration theory in practice [29]. The discriminant validity of SSP has been found to be >95% in identifying children with and without SP difficulties compared to the full 125-item *Sensory Profile* [30]. The SSP is recommended for research and screening due to the short administration time (15 minutes) [30], which is believed to heighten the potential response rate. The qualifications of the sensory profile and thereby also the SSP are based on assessments of performance from 1037 children without disabilities [31]. The Danish translation of the SSP was conducted by Pearson's Clinical Assessment group and a group of experienced Danish occupational therapists and a professional translator.

In addition to the SSP, the survey included a questionnaire about the following: demographic information (i.e., the child's age, sex, and municipality), whether the child participated in sports or gymnastics outside of school as an indication of physical activity, and the parent's educational level as an indication of the family's socioeconomic status. The parents' educational level was obtained using a hierarchical categorization four-level scale from the National Department of Education [32].

## 3. Data Analysis

Descriptive statistics were used to summarize the characteristics of the participants and to describe the prevalence of children with SP difficulties and the results in the different behavioral sections of the SSP. One-way ANOVA was used to examine differences between girls and boys regarding demographics (i.e., sports, geographic area, and parental level of education).

The relationship between sex and SPP scores in the different behavioral sections was explored using Chi^2^ analysis.

Logistic regression analysis was used to investigate possible associations between SP difficulties and the included demographics. The SSP scores were dichotomized into SSP scores within the category definite difference (i.e., SSP scores between 38 and 141), which was the dependent variable in the logistic regression analysis and higher SSP scores (i.e., SSP scores between 142 and 190 categorized as probable difference and normal SP performance). The independent variables included sex (girl or boy), participation in sports activities outside of school (no, once a week, or more than once a week), geographic area—municipalities were grouped according to different geographical regions in Denmark (i.e., Jutland, Zealand, or smaller Islands)—and parental level of education (mandatory or youth education, short academic, medium academic, or long academic education). When both parents' education level was known, the mean education level was used.

Data analysis was conducted using statistical software, IBM SPSS statistics version 25. The statistical significance level was set at *p* < 0.05.

## 4. Results

A total of 2043 participants answered the survey, 320 (15.7%) surveys were excluded because they did not complete all the SSP sections; the remaining 1723 responses were included in the analysis.

Demographic characteristics of the participants (*N* = 1723) are presented in Table 1. The children were between the ages of 5 and 11 years (mean age 7.32). There was no significant difference between boys and girls in the sample, in regard to the amount of sports outside of school, the different geographic areas of Denmark, or parental level of education.

Table 2 shows SSP results of the total sample; 56.3% of the children had an SSP score within the category *typical performance*, 22.4% had SSP scores in the category of *probable difference*, and 21.3% had an SSP score within the category *definite difference*. Scoring in the *definite difference* category suggests the child has SP difficulties. Among the 21.3% in the *definite difference* category, their SSP section scores were as follows: underresponsive/sensory-seeking behavior (32.7%), auditory filtering (31.1%), and visual/auditory sensitivity (8.1%).

Table 3 shows the results of SSP according to sex. A significantly higher percentage of boys (24.2%) than girls (17.8%) had an SSP score in the *definite difference* category (*p* ≤ 0.001). A difference was found between sex in all, especially in the section of *underresponsive/sensory-seeking behavior* (39.1% boys and 25.2% girls) and *auditory filtering* (35.1% boys and 26.2% girls).

Table 4 shows possible associations between the children's dichotomized SSP results and the included demographic factors. All investigated associations were statistically significant. Both the crude and adjusted associations showed a higher probability of SP difficulties among boys than girls (crude OR = 1.49, adjusted OR = 1.55). Adjusted associations showed a lower probability among children not participating in sports outside of school (OR = 0.55). Children of parents with a higher education level had a slightly higher probability of SP difficulties (adjusted OR = 0.80). Additionally, when adjusting for additional demographic factors, no association between SP difficulties and geographic area of where in Denmark the children attended school (adjusted OR = 1.00) was found.

## 5. Discussion

The aim of the study was to explore the prevalence of SP difficulties among Danish schoolchildren and explore possible associations between SP difficulties and demographic characteristics. The results of the study showed that 21% out of the 1723 children had SSP scores indicating SP difficulties. The study found an association between SP difficulties and sex—suggesting that boys are more likely than girls to have SP difficulties. Furthermore, an association was found between SP difficulties and participation in sports outside of school—suggesting higher probability of SP difficulties among children participating in sports. Due to the size of the study, the results seem generalizable.

The percentage of children with SP difficulties in this study was higher than the estimate of 5-16% from the United States [25] but was approximately the same as found in studies of Saudi (23%) [18] and Puerto Rico (20%) children [24] even though these were not otherwise comparable school systems. In accordance with the study from Puerto Rico, the present study found that the section of SSP where most children showed *definite difference* was underresponsive/sensory-seeking behavior (Danish = 32.7% and Puerto Rican = 38.3%). Underresponsive children seek out all kinds of movement activities without regard for personal safety. The frequent movement and sensory-seeking behavior can interfere with the child's ability to participate in activities [3]. This behavior may not only affect the individual child's ability to learn from school activities but may also disturb/distract other children in the classroom environment. In a 2016 study of elementary schoolchildren's off-task behavior, teachers identified that some of the most frequent reasons why a child stopped working on a task (of off-task behavior) were peers and self-distractions [33]. The off-task behavior was not identified as being due to SP difficulties but could be due to a variety of factors. Since these factors were not considered in the present study, such other factors may also have contributed to the children's underresponsive/sensory-seeking behavior.

The present study found that 31.1% (*n* = 536) of the children had an SSP score indicating *definite difference* in the SSP section regarding auditory filtering. Children who have difficulties with auditory filtering can be easily distracted and have difficulties functioning when there is a lot of noise around them, consequently leading to difficulty paying attention [3]. This may affect their ability to concentrate and learn from school activities, since there is usually noise in the classrooms. In Denmark, a typical class consists of one teacher and approximately 25 children; when considering the results of the number of children with sensory-seeking behavior combined with the number of children with difficulties regarding auditory filtering, it is thought to pose challenges to the classroom environment.

The results show that boys had a higher probability of having SP difficulties than girls (OR = 1.55, Cl: 1.22, 1.97). An Israeli study on children's preferred activities found that boys with SP difficulties showed a significantly lower preference for informal and skill-based activities than girls with SP difficulties [23]. However, the study found no significant difference in preferred activities between boys with or without SP difficulties, whereas it found that girls with SP difficulties showed a significantly greater preference for active physical activities than girls without SP difficulties [23]. This may indicate that even though the present study found a significantly higher percentage of boys with SP difficulties, the girls with SP difficulties might be more affected in their choices of activities. Hence, sex difference in SP should be studied further.

The present study found an association between SP difficulties and the children's participation in sports outside of school, whereas the children who participated in a sport were more likely to experience SP difficulties. Since this is a cross-sectional study, the direction of this association cannot be known, but it is found highly unlikely that participating in a sport should cause SP difficulties. However, what seems more likely is that children with SP difficulties and sensory-seeking behavior may be more likely to participate in structured physical activities, such as sports. Thus, the association between participating in structured physical activities and SP should be researched further.

Additionally, the study found that a higher parental education level slightly increased the probability of children having SP difficulties. Parental level of education was chosen as an indication of the family's socioeconomic status, and the findings were similar to the results from the Puerto Rican study, which found no significant link between income of parents and children with SP difficulties [24].

## 6. Methodological Considerations

Only 12 out of 94 municipalities participated in the study, and due to the data selection method, the response rate and possible sample size are not known. Still, the large sample size and the vast diversity of participants regarding age, geographic area of the municipalities, participation in sports or gymnastics outside of school, and parental level of education imply good representation.

This study used the Danish version of the SSP; even though the use of SSP in research is widespread, it does have some limitations. Measuring SP would have been strengthened by direct clinical observation of the children; however, this would have been difficult due to the large sample size. The use of the Danish version of SSP brings limitations, since this translated version has not been submitted to psychometric testing. Because of this, it is not certain that the instrument is as sensitive and precise in a Danish setting as it has been found to be in its original language. Additionally, the full 125-item Sensory Profile could have presented a more detailed description of the children's SP, but the disadvantage would probably have been a lower response rate and a smaller sample size, why the SSP seems to be the best choice given the purpose of the study.

Another limitation is the relatively high incompletion rate of the SSP (15.7%). Unfortunately, due to the anonymity of the participants, it was not possible to conduct a dropout analysis or research the reasoning behind this further. Perhaps, it was partly due to another limitation of the study, the recruitment process; the bulletin for participant recruitment and the questionnaire were only in Danish, which might have excluded parents who were not fluent in Danish. Participation in the study was also time-consuming, which could possibly have excluded parents with limited time, such as single parents. It was not possible for the parents to directly decline or give reasons for not participating in the study, so this assumption cannot be verified. To our knowledge, this is the first study of its kind in Scandinavia.

### 6.1. Implications

The study results suggest that approximately 20% of the children in Danish public schools might have SP difficulties and over 20% might be at risk of having SP difficulties. The results are thought to indicate that the school system could benefit from awareness of SP difficulties and addressing them to limit the potential for negative consequences for the children's further development and education. Focus on improving children's ability to process sensory input, perhaps through interventions such as sensory integration or tactile and proprioceptive stimulation, which can be provided through occupational therapy, could possibly facilitate the children's abilities to participate in and learn from school activities [10, 27].

## 7. Conclusion

The study found SSP scores that indicate that 21.3% of the children might have SP difficulties. The study found that the SSP section scores regarding underresponsive/sensory-seeking behavior (32.7%) auditory filtering (31.1%) were the most dominating. Additionally, the study found a significantly higher prevalence of SP difficulties in boys than girls. The school system could benefit from awareness of SP difficulties and interventions to support children participation in school activities.

## Figures and Tables

**Table 1:** Demographic characteristics of the children participating in the study (*N* = 1723).

	Girls	Boys	Total sample	*p* value^∗∗^
Sex^∗^	46.54% (*n* = 801)	53.22% (*n* = 916)	*n* = 1717	
Mean age, years (SD)	7.54 (SD: 1.19)	7.49 (SD: 1.23)	7.32 (SD: 1.21)	0.393

Sports or gymnastics^∗∗∗^				0.901
No	17.48% (*n* = 140)	22.38% (*n* = 205)	20.09% (*n* = 346)	
Once a week	45.57% (*n* = 365)	34.17% (*n* = 313)	39.49% (*n* = 679)	
More than once a week	36.95% (*n* = 296)	43.45% (*n* = 398)	40.42% (*n* = 696)	

Geographic area				0.519
Jutland	44.32% (*n* = 355)	44.43% (*n* = 407)	44.34% (*n* = 764)	
Zealand	32.08% (*n* = 257)	30.13% (*n* = 276)	30.99% (*n* = 534)	
Smaller islands	23.60% (*n* = 189)	25.44% (*n* = 233)	24.67% (*n* = 425)	

Parental level of education^∗∗∗∗^				0.784
Mandatory or youth education	17.25% (*n* = 265)	18.14% (*n* = 318)	17.72% (*n* = 585)	
Short academic	21.09% (*n* = 324)	21.22% (*n* = 372)	21.21% (*n* = 700)	
Medium academic	38.15% (*n* = 586)	38.39% (*n* = 673)	38.23% (*n* = 1262)	
Long academic	23.50% (*n* = 361)	22.25% (*n* = 390)	22.84% (*n* = 754)	

^∗^ *N* = 1717, six missing (mean age 7.51 years), not possible to identify sex. ^∗∗^Difference between girls and boys calculated with one-way ANOVA. ^∗∗∗^*N* = 1721, two missing, not possible to identify sports or gymnastics. ^∗∗∗∗^*N* = 3301, 145 missing, not possible to identify the education level of one parent.

**Table 2:** Short Sensory Profile scores (*N* = 1723).

	Typical performance % (*n*)	Probable difference % (*n*)	Definite difference % (*n*)
Total scores	56.3 (970)	22.4 (386)	21.3 (367)
Tactile sensitivity	72.1 (1243)	15.9 (274)	11.9 (206)
Taste/smell sensitivity	65.3 (1126)	17.9 (308)	16.8 (289)
Movement sensitivity	72.9 (1256)	16.6 (286)	10.5 (181)
Underresponsive/seeks sensation	43.9 (757)	23.3 (401)	32.8 (565)
Auditory filtering	47.1 (811)	21.8 (376)	31.1 (536)
Low energy/weak	73.6 (1269)	9.6 (166)	16.7 (288)
Visual/auditory sensitivity	79.8 (1375)	12.1 (208)	8.1 (140)

**Table 3:** Short Sensory Profile of girls and boys and comparison of definite difference in boys and girls (*N* = 1717).

	Typical performance % (*n*)		Probable difference % (*n*)		Definite difference % (*n*)		*p* value^∗^
	Girls	Boys	Girls	Boys	Girls	Boys	
Total scores	61.8 (495)	51.6 (473)	20.3 (163)	24.1 (221)	17.8 (143)	24.2 (222)	<0.001
Tactile sensitivity	74.6 (598)	70.0 (642)	14.4 (116)	17.0 (156)	10.8 (87)	12.8 (118)	0.057
Taste/smell sensitivity	64.6 (518)	66.1 (606)	20.9 (168)	15.1 (139)	14.3 (115)	18.6 (171)	0.002
Movement sensitivity	75.1 (602)	70.8 (649)	14.9 (120)	18.0 (165)	9.8 (79)	11.1 (102)	0.319
Underresponsive/seeks sensation	52.1 (418)	36.7 (337)	22.6 (181)	24.0 (220)	25.2 (202)	39.1 (359)	<0.001
Auditory filtering	54.1 (434)	40.9 (375)	19.6 (157)	23.9 (219)	26.2 (210)	35.1 (322)	<0.001
Low energy/weak	74.4 (596)	72.9 (668)	9.7 (78)	9.6 (88)	15.8 (127)	17.4 (160)	0.85
Visual/auditory sensitivity	80.4 (644)	79.1 (725)	10.8 (87)	13.2 (121)	8.7 (70)	7.6 (70)	0.45

Note: *n* = 1717, six missing, not possible to identify sex. ^∗^Comparison of girls' and boys' definite difference calculated with Chi^2^.

**Table 4:** Logistic regression analysis examining associations between SP difficulties and possible associated demographic factors (*N* = 1721).

Dependent variable was SSP scores indicating SP difficulties (i.e., the category of definite difference)						
Independent variable	Crude			Adjusted		
	OR	95% CI	*p*	OR	95% CI	*p*
Sex (ref. = girl)	1.49	1.18, 1.88	0.001	1.55	1.22, 1.97	<0.001
Sports or gymnastics outside of school (ref. = no)	0.53	0.45, 0.61	<0.001	0.55	0.47, 0.65	<0.001
Parental education level^∗^ (ref. = none/youth education)	0.77	0.69, 0.86	<0.001	0.80	0.71, 0.90	<0.001
Geographic's (ref. = Jutland)	0.85	0.73, 0.99	0.032	1.00	1.00, 1.01	0.008

^∗^ *N* = 3301. Mean education level was used when there were two parents. 141 were missing; not possible to identify education level.

## Data Availability

Due to the data being personal and given under a promise of anonymity, data is not published.
